# Genome shuffling of the nonconventional yeast *Pichia anomala* for improved sugar alcohol production

**DOI:** 10.1186/s12934-015-0303-8

**Published:** 2015-08-07

**Authors:** Guoqiang Zhang, Yuping Lin, Xianni Qi, Lixian Wang, Peng He, Qinhong Wang, Yanhe Ma

**Affiliations:** Key Laboratory of Systems Microbial Biotechnology, Tianjin Institute of Industrial Biotechnology, Chinese Academy of Sciences, 32 XiQiDao, Tianjin Airport Economic Area, Tianjin, 300308 China; University of Chinese Academy of Sciences, Beijing, 100049 China; Key Laboratory of Microbial Physiological and Metabolic Engineering, Institute of Microbiology, Chinese Academy of Sciences, Beijing, 100101 China

**Keywords:** Sugar alcohol, *Pichia anomala*, Nonconventional yeast, FACS, Genome shuffling

## Abstract

**Background:**

Sugar alcohols have been widely applied in the fields of food and medicine owing to their unique properties. Compared to chemical production, microbial production of sugar alcohols has become attractive because of its environmentally friendly and sustainable characteristics. Our previous study identified the nonconventional yeast *Pichia anomala* TIB-x229 as a potential producer of sugar alcohols from glucose. To further improve strain performance, we combined genome shuffling with optimized high throughput screening methods for the directed improvement of nonconventional yeast and complex phenotypes.

**Results:**

To accelerate strain improvement, a practical genome shuffling procedure was developed and successfully applied in the nonconventional yeast *P. anomala* to increase sugar alcohol production. Through two rounds of genome shuffling, an improved *P. anomala* isolate GS2-3 could produce 47.1 g/L total sugar alcohols from 100 g/L glucose, which was 32.3% higher than the original strain. In this process, a simple and accurate colorimetric assay was optimized and used for high throughput screening of sugar alcohol-producing strains. Moreover, a fluorescence-activated cell sorting method was developed to efficiently screen protoplast fusions for genome shuffling of nonconventional yeast.

**Conclusion:**

An efficient genome shuffling procedure was developed and applied to enhance the sugar alcohol production of the nonconventional yeast *P. anomala*. Our results provide a general platform for strain improvement of polyol-producing microorganisms or nonconventional microorganisms in the future.

**Electronic supplementary material:**

The online version of this article (doi:10.1186/s12934-015-0303-8) contains supplementary material, which is available to authorized users.

## Background

Sugar alcohols have attracted attention owing to their wide application in the food industry as food additives and in the chemical industry as commodity chemicals [1–3]. At present, many sugar alcohols depend on chemical methods under high temperature and pressure conditions for commercial manufacturing [4]. To provide an alternative pattern for sugar alcohol production, various sugar alcohol-producing microorganisms were screened and used for bioconversion. In our previous study, the nonconventional yeast *Pichia anomala* TIB-x229 was isolated and characterized for potential production of different functional sugar alcohols, such as d-arabitol, xylitol and ribitol [5]. However, the yield was not sufficiently high for the commercial process, and all attempts to enhance the yield by traditional optimization of the bioconversion process failed. Therefore, it was necessary to develop an efficient strategy to further improve the strain performance, which is an important step in the industrial commercial production process [6].

Although strain improvement has been achieved chiefly through classical mutation breeding and modern genetic engineering, such technologies are still limited by time-consuming processes with low productive mutation rates and multiplex gene modification, according to available information and research experiences [7–9]. By expanding the reach of shuffling technology from DNA fragments to the whole genome, genome shuffling provides an alternative means to classical strain improvement for accelerated evolution that requires no sequence information or tedious genetic tools. Genome shuffling was first applied to increase the production of the antibiotic tylosin in *Streptomyces fradiae* [10] and to improve the acid tolerance of *Lactobacillus* [11]. Recent studies have combined metabolic engineering and omics analysis with genome shuffling [12, 13] to further expand the scope of application. Therefore, genome shuffling has increasingly been used to rapidly improve different strains [14–16], especially for nonconventional organisms, such as *Zygosaccharomyces rouxii* [17] and *Hansenula anomala* [18]. However, this strategy depends largely on the efficiency of protoplast fusion and selection techniques. In laboratory studies of microbial genetics, two haploid strains with complementary genetic markers are fused and the hybrid cells can be identified by growth on selective media [19]. Many nonconventional strains, however, lack selectable genetic markers, making efficient identification of hybrids by genetic complementation difficult [20]. To overcome this problem, fluorescence-activated cell sorting (FACS) had been applied as an effective method in the development of improved industrial yeast strains [21–23]. In FACS, parent strains are first labeled with different fluorescent stains, and hybrids are then selected based on their dual fluorescence by flow cytometry (FCM).

In the present study, a newly developed genome shuffling was applied to rapidly improve the sugar alcohol production of *P. anomala*. The traditional random mutagenesis and efficient colorimetric screening method were combined to obtain mutants with subtle improvements in sugar alcohol production, and then, the positive populations were shuffled and selected by fluorescence-activated cell sorting. Finally, positive shuffled mutants showing significantly improved sugar alcohol production were further selected and identified. In general, the genome shuffling in our study is broadly useful for the rapid evolution of phenotypes in nonconventional microorganisms.

## Results and discussion

### Development of efficient colorimetric assay for sugar alcohol screening

Sugar alcohol-producing strains are usually screened and quantified by thin-layer chromatography (TLC), high-performance liquid chromatography (HPLC) and *p*-iodonitrotetrazolium violet (INT) methods [24–26]. However, these methods are time-consuming or suffer from high-cost and are limited for high throughput screening. Therefore, it is necessary to develop an efficient screening approach for sugar alcohol-producing microbes.

**Fig. 1:**
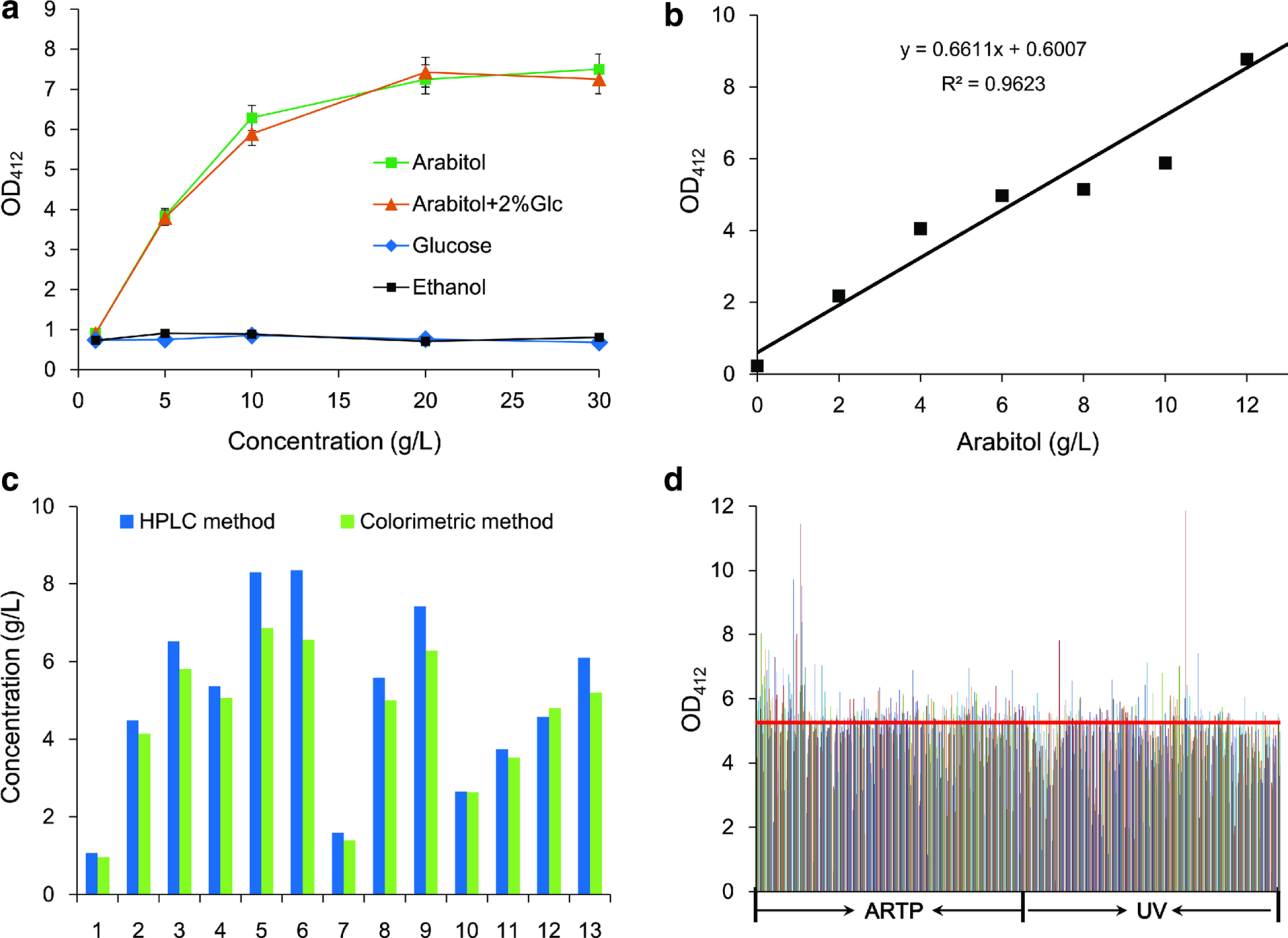
The construction of a colorimetric method for efficient sugar alcohol assay. **a** The interference test of the colorimetric method under different metabolites. **b** The standard curve of colorimetric method for d-arabitol detection. **c** Comparison of the colorimetric method with the HPLC method for sugar alcohol detection in different fermentation liquors. Data represent the average values of three independent experiments with deviation varying between 5 and 10% about the mean. **d** The construction of the *P. anomala* mutant library by ARTP and UV mutagenesis. The sugar alcohol production was preliminarily screened by colorimetric assay. The *red* *line* represents the sugar alcohol yield of the initial strain *P. anomala* HP by the colorimetric method.

In our study, a colorimetric method previously applied in trace detection of polyols [27, 28] was developed and optimized for the high throughput assay of sugar alcohols (Additional file 1: Fig. S1). d-arabitol was selected as a standard for the method construction because it is the main sugar alcohol product of *P. anomala*. By optimizing the reaction system, the standardized assay demonstrated a linear detection range of d-arabitol from 0 to 12 g/L. Although the linear relation was noticeably altered at 20 g/L sugar alcohol, the colorimetric curve was positively related with the sugar alcohol concentration and could be applied in the preliminary screening (Fig. 1a, b). To analyze effects of the substrate and by-products on sugar alcohol screening, an interference experiment was performed at different concentrations of glucose and ethanol (2–30 g/L). The results showed that glucose and ethanol had no interference in the quantitative analysis of sugar alcohols by the colorimetric method (Fig. 1a), which indicated that the developed assay is highly efficient for the determination of the content of sugar alcohol in biological samples. To gain a further understanding of the accuracy, the reference HPLC and the proposed colorimetric methods were applied to analyze sugar alcohol at different concentration levels. The results showed that the data measured by the colorimetric procedure agree with those determined by the reference HPLC method, and a regression line with an R^2^ of 0.9673 was obtained (Fig. 1c; Additional file 1: Fig. S1).

In this study, a convenient, reliable and low-cost colorimetric assay was developed for efficient primary screening and selection of strains with high productivity. The method is highly specific for sugar alcohols and can be performed on crude, non-purified extracts. The method uses low hazard and inexpensive reagents and requires only commonly available equipment. Finally, the method is sensitive and highly reproducible. Compared with HPLC and TLC methods, the colorimetric method facilitated sugar alcohol detection and made the operation of screening of sugar alcohol-producing strains more convenient. Although INT is another efficient method for sugar alcohol detection by specific enzyme catalysis, it is not suitable for high throughput assay because of the complex process and the expensive substrate *p*-iodonitrotetrazolium violet [29]. Therefore, the proposed colorimetric assay has clear advantages over the other methods and can be applied to high throughput screening for different polyol-producing strains.

### Development of a rapid hybrid cell selection procedure via FACS analysis

To achieve the efficient screening of hybrid cells without complementary genetic markers, FACS analysis based on fluorescent dyes was applied. In this process, hybrid cells are detected by carrying two dyes, and these cells can be analyzed and selected by FACS.

**Fig. 2:**
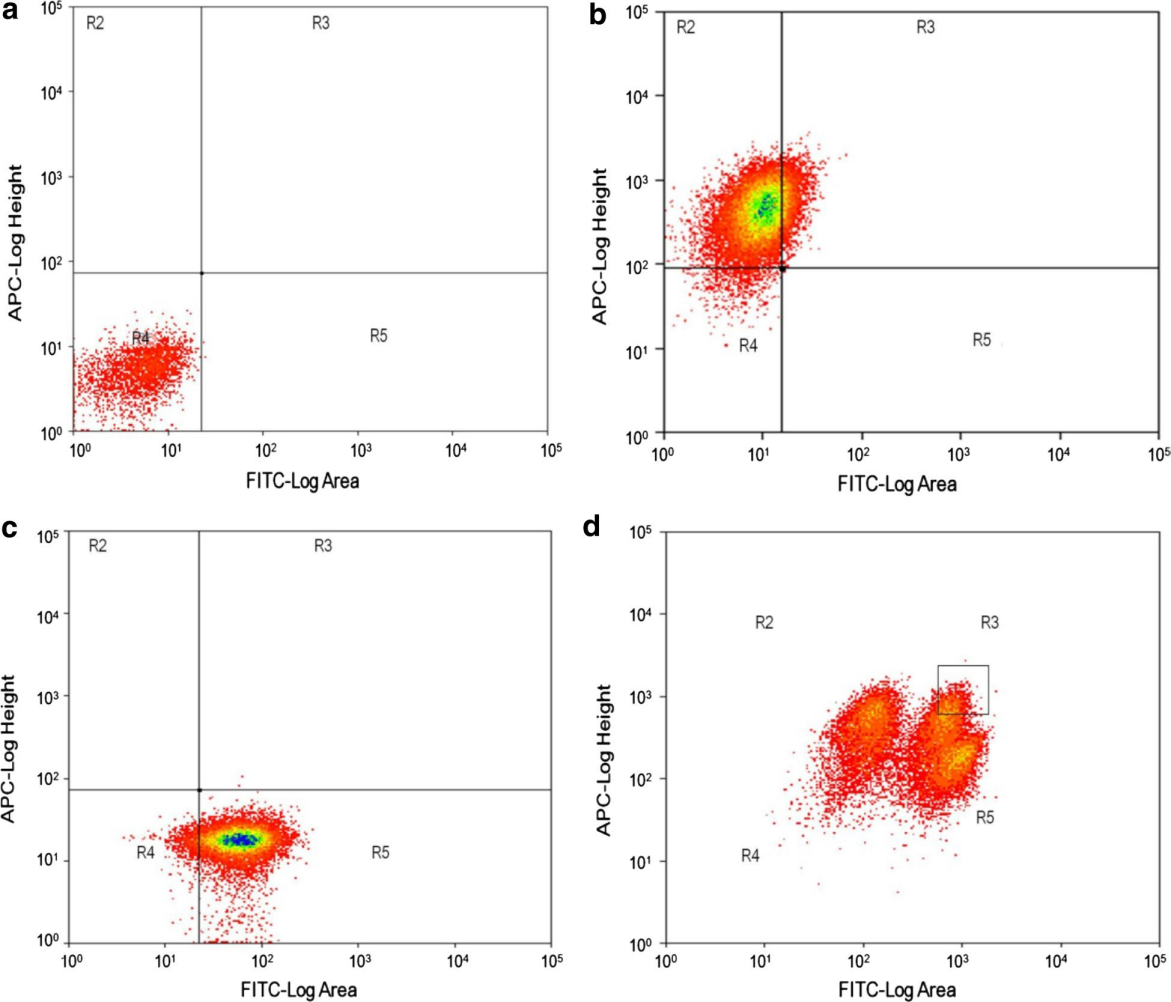
Flow cytometric analysis of the fluorescence distribution after protoplast staining and fusion. The parent and hybrids with different fluorescent dyes are represented as *dot plots* in the figure. Based on the different excitation and emission parameters, the sorting results were divided into four regions. R2 and R5 detected strains with single Nuclear Red and Nuclear Green, respectively. R3 detected possible hybrid strains with Nuclear Red and Nuclear Green. R4 was used as control to detect the blank strains. **a** Protoplasts of *P. anomala* without staining; **b** protoplasts of *P. anomala* stained by Nuclear Red; **c** protoplasts of *P. anomala* stained by Nuclear Green; **d** double-positive hybrid cells exhibiting high intensity fluorescence for Nuclear Red and Nuclear Green.

In this approach, parental protoplasts were prepared and then labeled with fluorescent dyes Nuclear Green and Nuclear Red, resulting in green and red fluorescence with laser excitation at 488 and 641 nm, respectively. After fusion, the hybrids were sorted by FCM, and the results are represented as dot plots (Fig. 2). As the control, strains without staining showed no fluorescence in the R4 gate (Fig. 2a). The parental strains showed single red and green fluorescence in different gates based on the staining with fluorescent dyes Red or Green (Fig. 2b, c). Overlap between the fluorescence regions of Green and Red was also observed, and possible compensation was performed. As shown in Fig. 2d, R3 is the sorting area showing cells that exhibit high intensity fluorescence with green and red and is identified as potential hybrid cells. In our study, some 2,500,000 protoplasts were rapidly sorted, and 15,300 potential hybrids were selected. Only approximately 1,000 colonies were found after incubation for regeneration; most protoplasts were not regenerated, probably because of damage during protoplast preparation, staining and laser sorting.

To facilitate screening and identification of the hybrid cells, different genetic markers were always necessary in previous studies, such as auxotroph [30] and drug resistance [31]. However, a genetic marker, such as auxotroph, affects the physiology and metabolism of the strain and leads to reduced performance in the production process. Furthermore, adding genetic markers to the parent strain is a difficult operation for some nonconventional strains. In this study the fluorescence-activated cell sorting was applied as a useful method for the hybrid cell selection of *P. anomala* without the need for genetic markers; in addition, this method is also available for the genome shuffling of other microbes. It may be possible to apply the technique for other native strains that are limited by unclear genetic backgrounds or unskilled genetic operations.

### The construction of a mutant library for genome shuffling by random mutagenesis

**Fig. 3:**
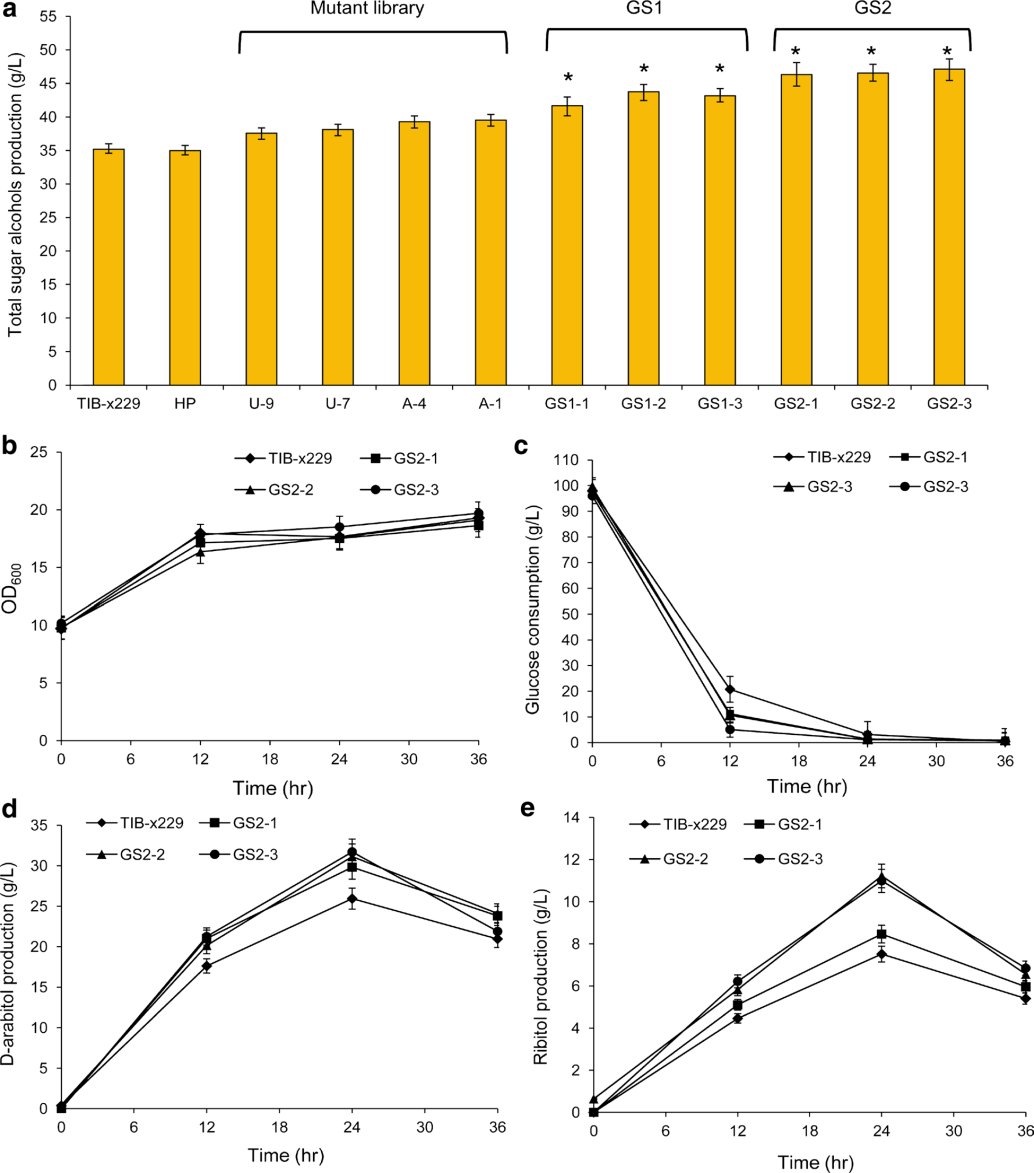
Comparison of bioconversion performance between the initial strain, mutants and shuffled strains. **a** Comparison of total sugar alcohols production between the initial strain, mutants and shuffled strains. **b**–**e** Comparison of growth condition, glucose consumption, d-arabitol production and ribitol production between initial strain and shuffled strains GS2-1, GS2-2 and GS2-3. U-: mutants obtained from five rounds of UV mutagenesis of *P. anomala* HP. A-: mutants obtained from five rounds of ARTP mutagenesis of *P. anomala* HP. GS1-: recombinants generated from the first round of genome shuffling. GS2-: recombinants generated from the second round of genome shuffling. Data represent the average values of three independent experiments with deviation varying between 5 and 10% about the mean. *Asterisk* indicates the significant difference in sugar alcohol production at *p* < 0.001 between TIB-x229 and the mutants based on ANOVA statistical test.

In the genome shuffling process, the wild type strain is usually treated by the traditional physical and chemical mutation methods, and the strains with superior performance are collected to form the parental library for the next step of recursive protoplast fusion [31, 32]. In this study, a haploid of sugar alcohol-producing *P. anomala* TIB-x229 [5] was first isolated and identified as *P. anomala* HP. The mutant library was constructed by ultraviolet (UV) and atmospheric and room-temperature plasma (ARTP) mutagenesis methods to generate genetic diversity. After the mutagenesis processes, mutants with the maximum sugar alcohol production were selected from approximately 2,000 mutants by colorimetric screening and were then prepared for the next round of mutation and screening. Through five rounds of continuous mutagenesis, a parent library with approximately 10,000 mutants was constructed and analyzed by the aforementioned colorimetric method (Fig. 1d). The sugar alcohol yield of the positive mutants was further confirmed by an HPLC method, and the four mutants (U-7, U-9, A-4 and A-1) showed clear superiority for sugar alcohol production. Compared to the initial *P. anomala* HP, the mutants U-7 and U-9 treated by UV had 7.3 and 8.9% improvement of sugar alcohol production. The yields of mutants A-4 and A-1 treated by ARTP were increased by 12.3 and 12.9%, respectively (Fig. 3a). These results showed that there was a slight improvement in mutants after several rounds of traditional mutagenesis. However, the single traditional mutagenesis was still a time-consuming process for strain engineering because of the low mutation rate and less diversity.

### Genome shuffling of *P. anomala* for improved sugar alcohol production

**Fig. 4:**
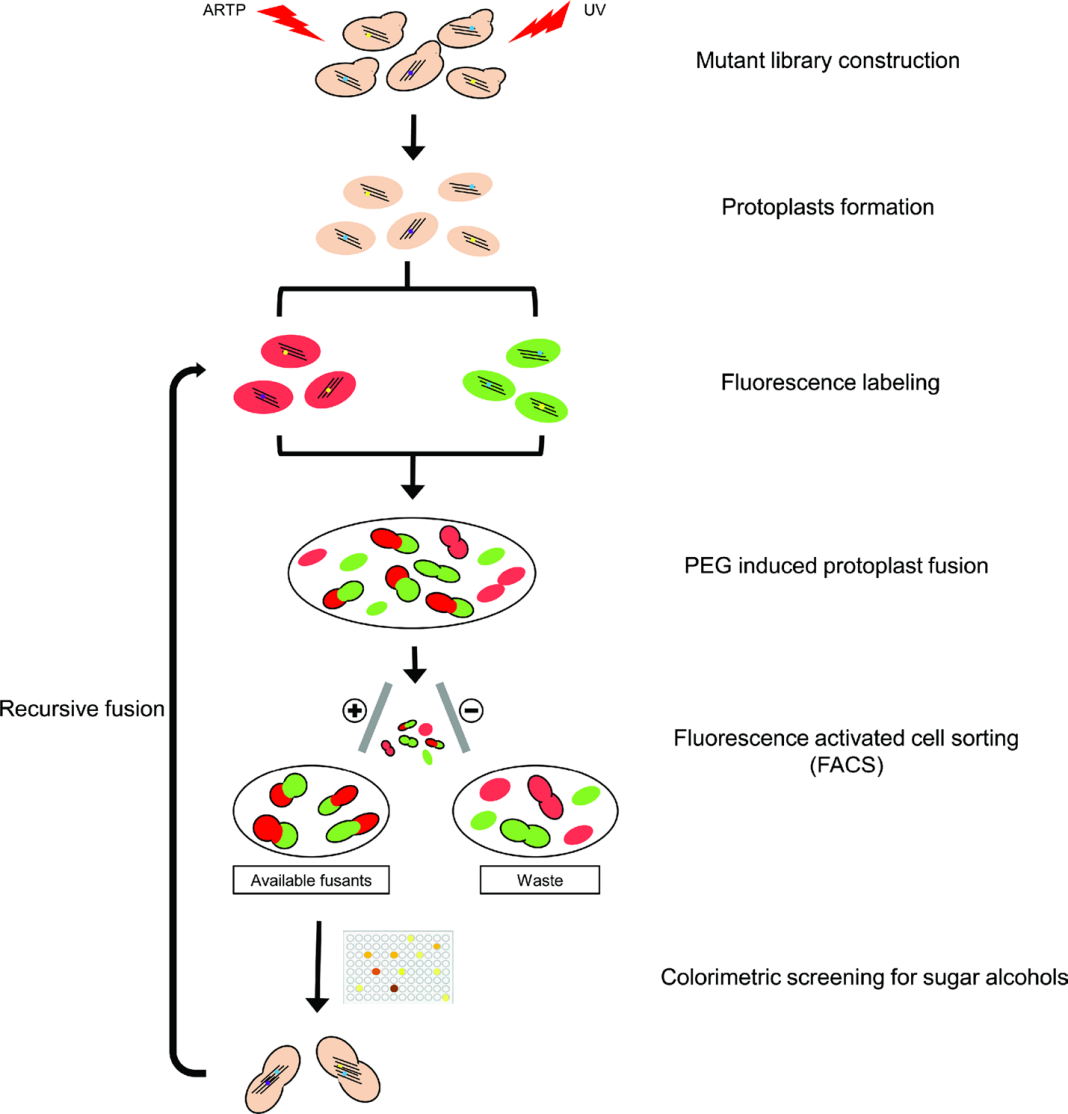
The procedure of genome shuffling for improved sugar alcohol production of *P. anomala.* The process includes six steps, such as mutant library construction, protoplast formation, fluorescence labeling, PEG induced protoplast fusion, FACS and colorimetric screening.

To further improve the performance of sugar alcohol productivity, the mutant strains (U-7, U-9, A-4 and A-1) with slightly improved performance were collected as the parental library for the next step of genome shuffling, which is a powerful means for rapid breeding of improved organisms without knowledge of the detailed genome information. To achieve the efficient screening for genome shuffling, the developed colorimetric assay of sugar alcohol and the FACS method were incorporated into the genome shuffling procedure for our nonconventional yeast *P. anomala* (Fig. 4).

The protoplasts were processed and fused by a chemical method induced by polyethylene glycol [33]. After the first protoplast fusion and screening by FACS, approximately 1,000 colonies with both red and green fluorescence were preliminarily cultivated and assayed for sugar alcohol production by colorimetric assay. The selected colonies exhibiting improved performance were further confirmed by HPLC. In the bioconversion process, d-arabitol and ribitol were produced from glucose by *P. anomala*. Compared to the parental strain *P. anomala* HP, three recombinants (GS1-1, GS1-2 and GS1-3) exhibited significantly improved productivity of total sugar alcohols by 19.5, 25.6 and 23.9%, respectively (Fig. 3a). The isolates GS1-2 and GS1-3 were used as the parent population for the following round of genome shuffling. Similarly, the resulting second-round isolates were further screened, and three isolates GS2-1, GS2-2 and GS2-3 were selected and evaluated and showed increased total sugar alcohol production of 46.1, 46.5 and 47.1 g/L, which was 29.5, 30.6, and 32.3% higher than that of parental strain *P. anomala* HP, respectively (Fig. 3a). We compared the relative DNA content between the parent strain and the shuffled strains by DAPI labeling and FCM (Additional file 1: Fig. S2). Compared to the parental strain *P. anomala* HP and the referenced haploid yeast *Saccharomyces cerevisiae* BY4741, the wild type TIB-x229, GS2-1, GS2-2 and GS2-3 strains had diploid DNA content. We assessed the performance and stability of shuffled strains through the bioconversion of sugar alcohols. For that purpose, bioconversion in sterile water containing 100 g/L glucose was used to compare the performance of the evolved strains, GS2-1, GS2-2 and GS2-3, with that of the original strain TIB-x229. Although the overall growth conditions were the same in all strains, the shuffled strains showed a slightly faster rate of glucose consumption (Fig. 3b, c). Likewise, the accumulation rate of d-arabitol and ribitol was higher in the shuffled strains. The yield of d-arabitol in the shuffled strains GS2-1, GS2-2 and GS2-3 was 0.29, 0.31 and 0.32 g/g, which was 11.5, 19.2, and 23.1% higher than that of the original strain *P. anomala* TIB-x229, respectively (Fig. 3d). The ribitol production in these shuffled strains was 8.46, 11.23 and 10.98 g/L (Fig. 3e), which was also slightly higher than that in the original strain (7.51 g/L). These results showed that the improvement of the shuffled strains in sugar alcohol production was due to the accumulation of d-arabitol and ribitol. In this study, two rounds of genome shuffling achieved efficient gains in sugar alcohol yield. The results further indicated that genome shuffling is a much more powerful means for breeding improved organisms, especially for those strains that have undergone classic strain improvement many times.

In recent years, there are also other different reports on sugar alcohols improvement including metabolic engineering [34], natural screening [5], fermentation optimization [35] and mutation breeding [36]. However, there was not any study reported about improving performance of sugar alcohol-producing strains by genome shuffling, because there were some obstacles in this process, such as a lack of efficient sugar alcohol-detection methods and available yeast selective markers. In our study, we developed the practicable genome shuffling for sugar alcohol-producing strains by combining the colorimetric assay and fluorescence-activated cell sorting, which provided a more efficient way for sugar alcohol-producing strain improvement.

## Conclusion

In this study, we have developed a feasible genome shuffling strategy for nonconventional sugar alcohol-producing yeast *P. anomala* including a colorimetric assay for rapid sugar alcohol screening and fluorescence-activated cell sorting for efficient hybrid cell selection. After two rounds of shuffling, we obtained an evolved *P. anomala* strain GS2-3 exhibiting the highest yield of sugar alcohols from glucose. Besides, the developed genome shuffling procedure has a significant potential for further application in other natural and nonconventional microorganisms.

## Methods

### Materials

Yeast extract and tryptone were procured from OXOID (Hampshire, UK). Agar powder and snailase were purchased from Solarbio Science Technology Co., Ltd (Beijing, China). Glucose, d-arabitol and other standard samples were purchased from Sigma (St. Louis, MO, USA). Nuclear Green LCS1 and Nuclear Red LCS1 for fluorescence staining were purchased from AAT Bioquest (Sunnyvale, CA, USA). Polyethylene glycol (PEG) 6000, KIO_4_ and other chemicals were purchased from Sinopharm (Beijing, China). Hypertonic buffer (HB) consisted of 0.01 M Tris–HCl, pH 6.8, 20 mM MgCl_2_, and 0.5 M sucrose as a stabilizer for protoplasts. Nash reagent was freshly prepared by mixing 150 g of ammonium acetate, 2 mL of glacial acetic acid, and 2 mL of pentane-2,4-dione and bringing the mixture to 1 L with distilled water.

### Strains and culture conditions

The sugar alcohol-producing yeast *P. anomala* TIB-x229 (CGMCC No. 5482) was used as the initial strain in this study. The recombinant yeast GS2-3 was preserved in the China General Microbiological Culture Collection Center as *P. anomala* TIB G2-3 (CGMCC No. 10260). The yeast strain was maintained on YPD agar plates with 10 g/L yeast extract, 20 g/L tryptone, 15 g/L agar, and 20 g/L glucose and was incubated at 30°C. The inoculums were prepared in YPD media, and the composition was 10 g/L yeast extract, 20 g/L tryptone, and 20 g/L glucose. The inoculants were incubated by placing the test tubes on a reciprocal shaker and shaking at 200 rpm at 30°C for 12 h. The protoplast was maintained on hypertonic YPD (HYPD) medium for cell regeneration.

### Optimization of the efficient colorimetric screening method for sugar alcohol

In this method, sugar alcohol was first oxidized and generated formaldehyde under acidic periodate conditions (pH 1.0). The residual periodate was reduced by the addition of l-rhamnose. The formaldehyde was then determined colorimetrically with Nash reagent, which produced yellow 3,5-diacetyl-1,4-dehydrolutidine with a maximum absorption at 412 nm (Additional file 1: Fig. S1a). The specific operation was as follows: culture containing sugar alcohols was centrifuged at 10,000×*g* for 10 min and 20 μL supernatant was placed in 96-deep well plates by a multiple channel receiver, and 500 μL of 0.015 M KIO_4_ in 0.12 M HCl was added. After being mixed and then allowed to stand for 10 min at room temperature, 400 μL of a 1% l-rhamnose solution was added to remove excess periodate. The color was developed for 20 min in an incubator at 63°C after adding 600 μL of Nash reagent. After cooling, the absorbance was measured at 412 nm with a spectrophotometer.

### Construction of the parent mutant library

The haploid yeast *P. anomala* HP was isolated from *P. anomala* TIB-x229 on McCLary medium containing 1 g/L glucose, 1.8 g/L KCl, 2.5 g/L yeast extract, 8.2 g/L NaAc and 15 g/L agar, according to the previous method [37]. UV and ARTP were used as mutation methods to achieve the initial mutant library, as previously described with slight modifications [38, 39]. For UV treatment, the liquid cultures spread on YPD plates were placed under a preheated 15 W UV lamp at a vertical distance of 20 cm and were irradiated for 100 s to achieve a survival rate of 10%. The operating parameters of the ARTP apparatus (Siqingyuan Biotechnology, Wuxi, China) were as follows: (1) pure helium was used as the plasma working gas at 10 L/min; (2) the radio frequency power input was 115 W; and (3) the distance between the plasma torch nozzle exit and the sample plate was 2 mm. In ARTP mutagenesis, 10 μL of fresh cell suspension was evenly spread on a sterilized steel plate and exposed to the airflow for 90 s. After treatment, the dry cells were eluted with 500 μL of sterile water into a new tube, and 200 μL of the liquid was spread on YPD medium. Colonies were inoculated into deep-well microplates and cultivated at 30°C/800 rpm. After incubation for 12 h, the whole cells were harvested by centrifugation at 3,000×*g* for 3 min. The whole cells were suspended in 100 g/L glucose solution, and bioconversion was performed for 12 h. Using the above mentioned colorimetric screening method, mutants with the highest sugar alcohol yield were selected for the next mutation. The mutant library was subjected to sequential mutagenesis five times and was screened by the colorimetric method. Finally, the four positive mutants with the highest sugar alcohol yield were confirmed by the HPLC method and were prepared for genome shuffling.

### The FACS analysis of hybrid cells based on fluorescent dyes

Yeast mutants were cultured at 30°C for 12 h in 10 mL of YPD. Cells were harvested by centrifugation, washed twice with distilled water and incubated in HB containing 0.02 M β-mercaptoethanol for 20 min at 30°C. Cells were collected and re-suspended in HB containing 2% (w/v) snailase for enzymatic digestion of the cell wall. The cell suspension was incubated in a water bath at 30°C for 60 min. Cells were washed twice and suspended in HB for genome shuffling. The efficiency of protoplast formation was determined by microscopy.

An equal number of protoplasts from different populations of mutants were divided equally into two parts. The two parts were stained with 0.25 μM nuclear Green and 0.1 μM Nuclear Red for 30 min, respectively. The stained protoplasts were washed twice by HB and were resuspended in HB containing 40% (v/v) polyethylene glycol (PEG6000) and 0.01 M CaCl_2_. After gently shaking for 15 min at 30°C to allow protoplast fusion, the fused protoplasts were centrifuged, washed and resuspended in HB. The double-positive hybrid cells with red and green fluorescent were selected by a Beckman MoFlo XDP flow cytometer (Brea, CA, USA). Fluorescence was monitored in fluorescence channels FL1 and FL8 with an appropriate laser. The selected hybrid cells were collected and cultivated on HYPD medium. After cultivating at 30°C for 48 h, the sugar alcohol productivity of the strains was screened by the efficient colorimetric method previously described. After preliminary screening, the sugar alcohol production of the selected strains was further analyzed by HPLC, and the three mutants with the highest production were selected as the starting strains for the next genome shuffling. Two successive rounds of protoplast fusion were performed.

### Determination of ploidy by FCM

FCM analysis of DNA content was performed according to Andalis et al. [40]. Briefly, exponentially growing cells were collected and washed with ice-cold sterile water and fixed with 70% ethanol at 4°C for 30 min. Cells were collected again, washed two times and resuspended in ice-cold sterile water containing a final concentration of 1 μg/mL 4′,6-diamidino-2-phenylindole (DAPI). Samples were incubated at room temperature in the dark for 30 min. The fluorescence intensity was measured via a Beckman MoFlo XDP flow cytometer.

### The evaluation of the shuffled strains for sugar alcohol production

A single colony was inoculated into 20 mL of YPD medium in a 250 mL shake flask and cultured at 30°C/200 rpm. After incubation for 12 h, the whole cells were harvested by centrifugation at 3,000×*g* for 3 min. After washing twice with distilled water, whole cells were suspended in 100 g/L glucose solution and the final OD_600_ of bioconversion system was controlled at ~10. The bioconversion was performed at 30°C/250 rpm in a 250 mL shake flask. Aliquots of culture filtrate collected at the same time interval were centrifuged at 10,000×*g* for 10 min. The concentrations of glucose and sugar alcohols were measured by HPLC (Agilent, Santa Clara, CA, USA) equipped with an Hi-Plex Ca column (7.0 × 300 mm, Agilent, Santa Clara, CA, USA). The column was eluted with H_2_O at a constant rate of 0.6 mL/min at 78°C. An Agilent 1260 refractive index detector (Agilent, Santa Clara, CA, USA) was used. The resulting chromatograms were compared to the chromatograms of the known standards and calibration curves for identification and quantification of the sugar alcohols. Sugar alcohols quantified in at least three biological replicates were subjected to the ANOVA-based statistical test, and those with *p*-values <0.001 and fold-changes >1.2 were considered significantly changed.

## Additional file

Additional file 1:
**Fig. S1.** The colorimetric assay of sugar alcohols. **a** The flow chart of the colorimetric method for sugar alcohol screening. **b** The correlation of the two sugar alcohol-detection methods by linear regression. H and C represent the HPLC and colorimetric methods, respectively. **Fig. S2.** Comparison of the DNA content among the parent and shuffled strains, as determined by flow cytometry. The DNA content is shown for a haploid control strain *S. cerevisiae* BY4741, haploid parent strain *P. anomala* HP, diploid strain *P. anomala* TIB-x229 and shuffled strains GS2-1, GS2-2 and GS2-3.

## Abbreviations

UV: ultraviolet
ARTP: atmospheric and room-temperature plasma
HPLC: high-performance liquid chromatography
TLC: thin-layer chromatography
INT: *p*-iodonitrotetrazolium violet
FACS: fluorescence-activated cell sorting
OD_412_: optical density at 412 nm
FCM: flow cytometry
CGMCC: China General Microorganism Culture Center
