# Supplementary material for: Genome shuffling of the nonconventional yeast Pichia anomala for improved sugar alcohol production
Source: Microb Cell Fact. 2015 Aug 7;14:112. doi: 10.1186/s12934-015-0303-8 (PMC4527335; doi:10.1186/s12934-015-0303-8)
Supplement: Additional file 1: — Fig. S1. The colorimetric assay of sugar alcohols. a The flow chart of the colorimetric method for sugar alcohol screening. b The correlation of the two sugar alcohol-detection methods by linear regression. H and C represent the HPLC and colorimetric methods, respectively. Fig. S2. Comparison of the DNA content among the parent and shuffled strains, as determined by flow cytometry. The DNA content is shown for a haploid control strain S. cerevisiae BY4741, haploid parent strain P. anomala HP, diploid strain P. anomala TIB-x229 and shuffled strains GS2-1, GS2-2 and GS2-3. [file 12934_2015_303_MOESM1_ESM.docx]

**Supplementary Information**

**Figure S1**


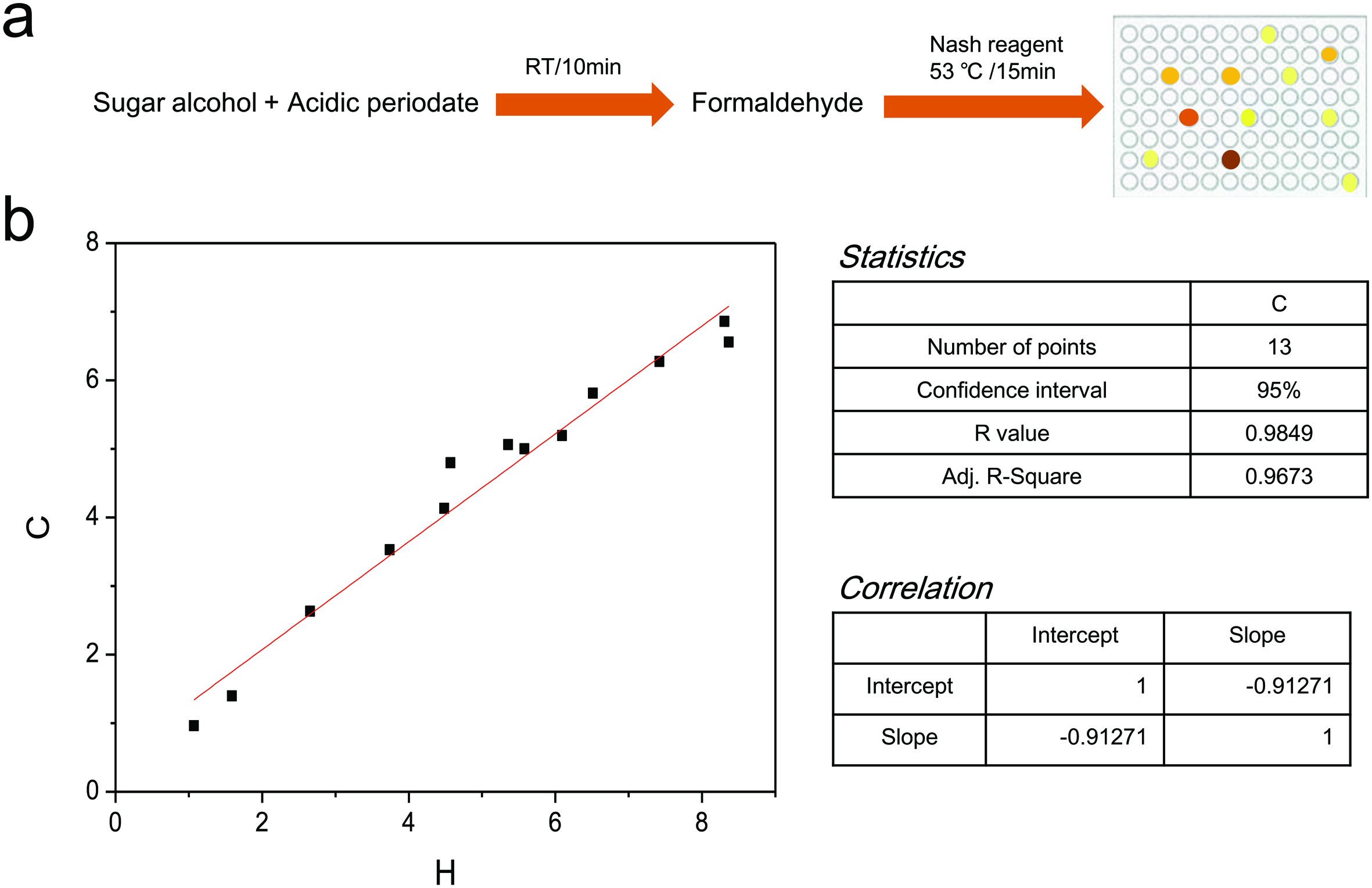


**Figure S1** The colorimetric assay of sugar alcohols. **a.** The flow chart of the colorimetric method for sugar alcohol screening. **b.** The correlation of the two sugar alcohol-detection methods by linear regression. H and C represent the HPLC and colorimetric methods, respectively.

**Figure S2**


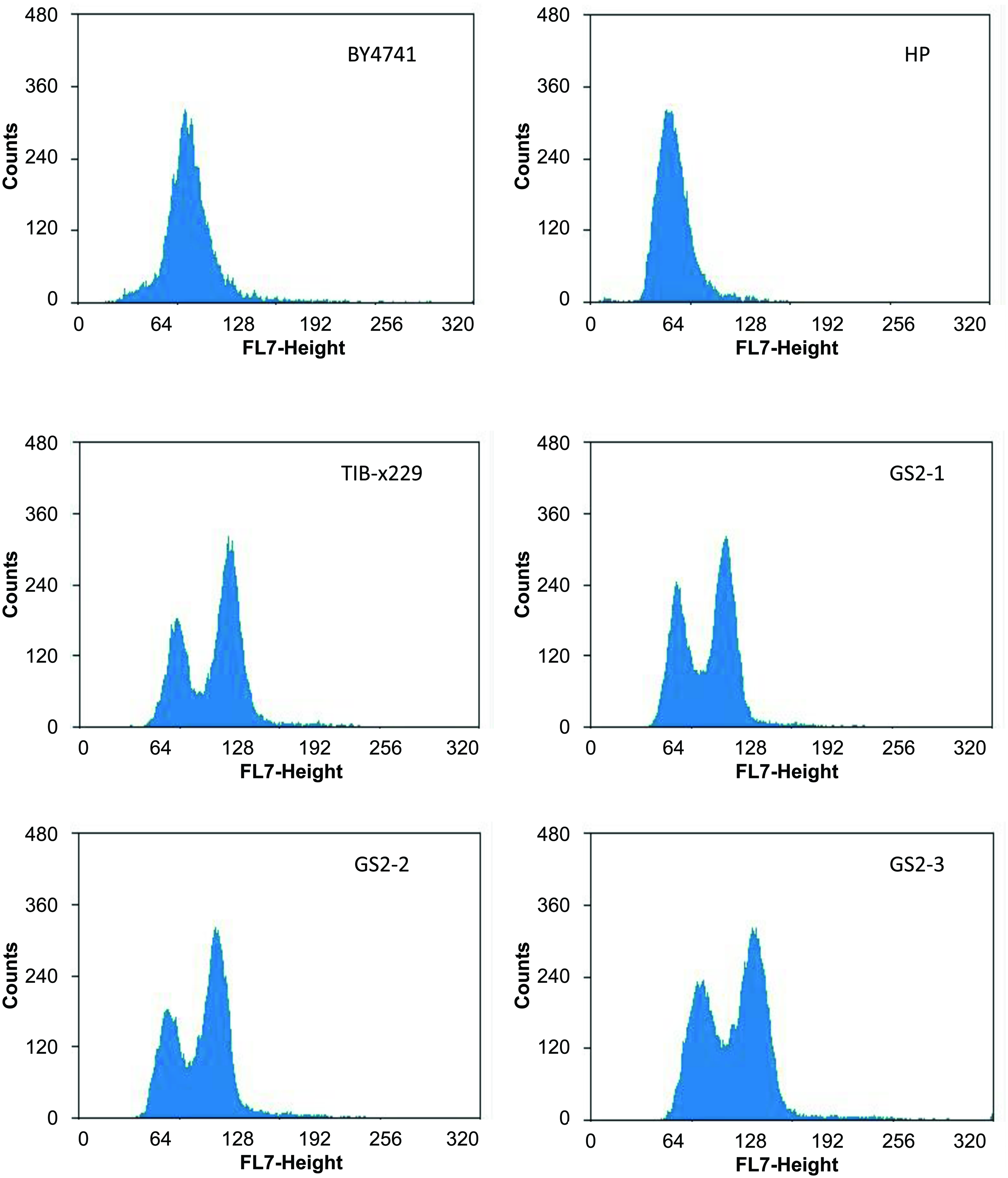


**Figure.S2** Comparison of the DNA content among the parent and shuffled strains, as determined by flow cytometry. The DNA content is shown for a haploid control strain *S. cerevisiae* BY4741, haploid parent strain *P. anomala* HP, diploid strain *P. anomala* TIB-x229 and shuffled strains GS2-1, GS2-2 and GS2-3.
